# IBSP, a potential recurrence biomarker, promotes the progression of colorectal cancer via Fyn/β‐catenin signaling pathway

**DOI:** 10.1002/cam4.3959

**Published:** 2021-05-13

**Authors:** Yan Chen, Ying Qin, Mengmeng Dai, Liping Liu, Yong Ni, Qinsheng Sun, Lulu Li, Yaoyao Zhou, Cheng Qiu, Yuyang Jiang

**Affiliations:** ^1^ School of Life Sciences Tsinghua University Beijing China; ^2^ State Key Laboratory of Chemical Oncogenomics Key Laboratory of Chemical Biology Tsinghua Shenzhen International Graduate School Shenzhen Guangdong China; ^3^ National & Local United Engineering Lab for Personalized Anti‐tumor Drugs Shenzhen Kivita Innovative Drug Discovery Institute Tsinghua Shenzhen International Graduate School Shenzhen Guangdong China; ^4^ Department of Gastrointestinal Surgery Shenzhen Second People's Hospital Shenzhen Guangdong China; ^5^ Department of Hepatobiliary and Pancrease Surgery Shenzhen People's Hospital Shenzhen Guangdong China; ^6^ Department of Hepatopancreatobiliary Surgery Shenzhen Second People's Hospital Shenzhen Guangdong China

**Keywords:** colorectal cancer, diagnosis and treatment, Fyn/β‐catenin signaling pathway, integrin‐binding sialoprotein (IBSP), metastatic recurrence

## Abstract

Colorectal cancer (CRC) is a frequently occurring digestive system cancer and postoperative tumor metastasis and recurrence are the main reasons for the failure of CRC treatment. The aim of this study was to identifying and validating key genes associated with metastatic recurrence of CRC. RNA expression of three datasets (GSE17538, GSE32323, and GSE29623) was used for biomarker discovery. We identified integrin‐binding sialoprotein (IBSP) as a candidate biomarker which was validated in three clinical cohorts (GSE41258, GSE21510, and GSE39582) and our clinical specimens. The results suggested that IBSP expression significantly increased at mRNA and protein levels among CRC cases, which was associated with metastatic recurrence, metastasis, high risk of recurrence, and poor survival in CRC. Consistent results were obtained in CRC cells. The relative level of serum IBSP evidently increased among CRC patients relative to normal controls, and downregulated after operation. As suggested by gene set enrichment analysis (GSEA), the IBSP level was associated with cell‐matrix adhesion in CRC. Functional experiments in vitro showed that IBSP promoted the growth and aggressiveness of CRC, and the potential mechanism by which IBSP promoted carcinogenesis of CRC was the abnormal activation of Fyn/β‐catenin signaling pathway. To sum up, findings in the present work indicate that IBSP can serve as the candidate biomarker for the diagnosis, treatment, and prognosis of CRC.

## Graphical abstract

In this study, we identified and validated IBSP as a key gene associated with metastatic recurrence of CRC which might serve as the candidate biomarker for the diagnosis, treatment, and prognosis of CRC. Our results suggested that IBSP expression significantly increased at mRNA and protein levels among CRC cases, which was associated with metastatic recurrence, metastasis, high risk of recurrence, and poor survival in CRC. Functional experiments in vitro showed that IBSP promoted the growth and aggressiveness of CRC, and the potential mechanism by which IBSP promoted carcinogenesis of CRC was the abnormal activation of Fyn/β‐catenin signaling pathway.

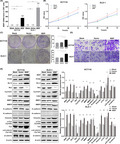

## 1 INTRODUCTION

Colorectal cancer (CRC) is one of the malignancies of digestive system and accounted for 6.1% of global cancer incidence and 9.2% of global cancer mortality in 2018.^1^ At present, surgery is still the only way to cure CRC. Although the level of surgical treatment has made great progress, the 5‐year survival rate of CRC remains unsatisfactory. Postoperative tumor metastasis and recurrence are the main reasons for the failure of CRC treatment.^2^ CRC usually relapse in 5% stage I cases, 10%–20% stage II cases, and 30%–45% stage III cases, even though radical surgery has been performed.^3^, ^4^ As suggested by existing studies, some factors exert vital parts in CRC relapse and metastasis, such as tumor location, clinical‐pathologic stratification, K‐ras mutations, microsatellite instability, mismatch repair genes, COX‐2 expression, circulating tumor DNA, gut microbiota, and so on.^5^, ^6^, ^7^, ^8^, ^9^, ^10^ However, these factors do not exhibit adequate reliability for predicting recurrence. Therefore, there remains an urgent to appraise valuable clinical entities and biomarkers aiming to identify patients at risk of recurrence.

The present work utilized three GEO database‐derived gene expression profiles to discover the differentially expressed genes (DEGs) in relapsed versus non‐relapsed cases. Three significantly upregulated genes (CYP1B1, COMP, and IBSP) were found between the recurrence versus non‐recurrence groups (> 1.5‐fold change, and adjusted *p* < 0.05). We selected IBSP, whose clinical significance in CRC remains unclear, yet it has attracted wide attention in tumor research for this study. IBSP belongs to the small integrin‐binding ligand, N‐linked glycoprotein (SIBLING) family, and it is located on chromosome 4q21.1.^11^, ^12^ IBSP is responsible for encoding a secretory glycoprotein that contains 317 amino acids and is mostly detected within bone tissues.^13^ The glycoprotein can combine with hydroxyapatite along with calcium through the acidic amino acid clusters; at the same time, it can regulate cell attachment by the arginylglycylaspartic acid (RGD) sequence recognizing vitronectin receptor.^14^, ^15^, ^16^ As far as CRC is concerned, cell attachment exerts a critical part in its metastasis capacity, which can thereby regulate its prognostic outcome. IBSP has been recognized to be the possible oncogene, which is usually overexpressed and upregulated in numerous cancers, such as prostate cancer (PC), breast cancer (BC), and lung cancer (LC).^17^, ^18^, ^19^, ^20^ Only few studies have reported the relationship between IBSP and CRC, and the biological function and molecular mechanism of IBSP involved in CRC progression are completely unknown. The present work measured IBSP expression within CRC samples at mRNA and protein levels through qRT‐PCR together with Western blotting analysis. Meanwhile, we analyzed whether inhibiting or upregulating IBSP level affected the CRC cell malignant behaviors and examined the mechanism by which IBSP promoted the carcinogenesis of CRC, as well as the relevant clinical value.

## 2 MATERIALS AND METHODS

### 2.1 Biomarker discovery analysis

To obtain the key genes related to metastatic recurrence in CRC, we initially analyzed the GSE17538 dataset comprising of 173 CRC patients (35 with recurrence and 138 without recurrence), the GSE32323 dataset comprising of 18 CRC patients (8 with metastasis or metastatic recurrence and 10 without recurrence), and the GSE29623 dataset comprising of 53 CRC patients (9 with recurrence and 44 without recurrence). We selected upregulated genes in the recurrence group versus the non‐recurrence group as candidate genes. Four R language packages (“Affy,” “limma,” “ggplot2,” and “VennDiagram”) were used to standardize the chips data, get and visualize the common upregulated genes in all the three datasets. With the characteristics of fold change>1.5, adjusted *p* value <0.05, genes were considered to be significantly upregulated and may take an important part in metastatic recurrence of CRC. IBSP was selected for further evaluation, and we also verified the IBSP expression difference among normal colonic mucosa, primary, and metastatic CRC in GSE41258 dataset, between CRC patients with non‐recurrence and metastatic recurrence in GSE21510 dataset, and between CRC patients with high risk and low risk of recurrence in GSE39582 dataset.

### 2.2 CRC clinical samples and cell lines

This study obtained altogether 82 primary CRC samples, 5 CRC liver metastatic recurrence samples, along with the corresponding non‐carcinoma samples (5 cm from tumor edge) from CRC patients receiving surgery at the Shenzhen Second People's Hospital and Shenzhen People's Hospital. The 82 CRC patients who provided primary tumor tissues were all initially diagnosed, without family genetic history, and had not received radiotherapy or chemotherapy before surgery. Metastatic recurrence in liver occurred after the operation in five relapsed patients. After resection, the samples were preserved within the RNAlater™ stabilization solution (Thermo Fisher Scientific) at once, followed by preservation under −80°C until later use. Peripheral blood samples (including 82 preoperative and 46 postoperative samples from CRC patients, together with 82 from normal controls) were sampled into the clot activator‐free vacuum blood tubes, followed by 2 h of clotting under 4°C and 10 min of rotation at 2000 rpm under 4°C. Thereafter, serum samples were added to the RNase‐free tubes and later preserved under −80°C. The clinicopathological parameters of the sample were shown in Table 1. None of the primary CRC cases received preoperative treatment. Human colon adenocarcinoma HCT116 cells and Human normal colorectal epithelial FHC cells were purchased from the ATCC. The human colon adenocarcinoma cell lines HT‐29, DLD‐1, LS174 T, and SW480 were purchased from Cell Bank, China Academy of Sciences. Each cell line was then cultivated in line with specific protocols.

**TABLE 1:** Relationship between IBSP expression and clinicopathological parameters of 82 CRC patients

Number of patients (N%)		IBSP expression levels		Ratio (High/Low)	*p* value
		High expression	Low expression		
Gender					
Male	46 (56.1%)	11	35	0.314	0.587
Female	36 (43.9%)	7	29	0.241	
Age					
<50	24 (29.27%)	4	20	0.2	0.793
≥50	58 (70.73%)	14	44	0.318	
TNM stage					
I+II	37 (45.12%)	5	32	0.156	0.046*
III+IV	45 (54.88%)	13	32	0.406	
Lymph nodes metastasis					
N0	37 (45.12%)	5	32	0.156	0.046*
N1+N2	45 (54.88%)	13	32	0.406	
Distant metastasis					
M0	70 (85.36%)	15	55	0.273	0.244
M1	12 (14.64%)	3	9	0.333	

N0: No regional lymph node metastasis; N1: Metastasis in 1–3 regional lymph nodes; N2: Metastasis in four or more regional lymph nodes.

M0: No distant metastasis; M1: Distant metastasis.

* There is significant difference.

### 2.3 Knockdown or overexpression of IBSP in CRC cells

The Lipofectamine 3000 Reagent (Invitrogen) was used to transfect the anti‐IBSP small interfering RNA or negative control (GenePharma) and IBSP‐overexpressing plasmid or vector control plasmid (pCDNA3.0, GENEWIZ) to CRC cells following specific protocols. The siRNA and negative control sequences were shown in Table 2. Cells were collected for further experiments at 48 h after transfection.

**TABLE 2:** IBSP‐siRNA and negative control sequences

	Sense (5'−3')	Antisense (5'−3')
IBSP‐siRNA1	GCCUAUGAAGAUGAGUACA	UGUACUCAUCUUCAUAGGC
IBSP‐siRNA2	GGCACCUCGAAGACAACAA	UUGUUGUCUUCGAGGUGCC
IBSP‐siRNA3	GGAAUGGCCUGUGCUUUCU	AGAAAGCACAGGCCAUUCC
Negative control	UUCUCCGAACGUGUCACGU	ACGUGACACGUUCGGAGAA

### 2.4 RNA extraction from tissues, cells, and qRT‐PCR

TRIzol reagent (Thermo Fisher Scientific) was adopted for extracting total tissue and cellular RNA. The BeyoRT™ II thesis kit was used in combination with the gDNA Eraser (Beyotime Bio Inc) for the synthesis of cDNA through the reverse transcription of 1 µg RNA. Then, the 7500 PCR system (Thermo Fisher Scientific) was adopted for qRT‐PCR analysis and the procedure was completed under the following conditions: 5 min under 95°C; followed by 10 s under 95°C and 35 s under 60°C for 40 cycles (Takara Bio). The primer sequences used were shown in Table 3. GAPDH served as the endogenous reference and the IBSP mRNA level was determined by 2^−△△Ct^. Each experiment was conducted three times.

**TABLE 3:** Primer sequences used for qRT‐PCR

Gene	Sequence
GAPDH‐F GAPDH‐R	GAGTCAACGGATTTGGTCGT TTGATTTTGGAGGGATCTCG
IBSP‐F IBSP‐R	AAGGGCACCTCGAAGACAAC CCCTCGTATTCAACGGTGGT
FYN‐F FYN‐R	CCTCGAGAATCCCTGCAGTT GCGCTTCCTCAAGGAATGAT
LCK‐F LCK‐R	CACGCTGCTCATCCGAAATG GGTTGTCTTGCAGTGGGGAA
LYN‐F LYN‐R	ATGTGAGAGATCCAACGTCCAA AAAAGCTGCCTTTCTGCGTC

### 2.5 Western blotting

The radioimmunoprecipitation assay (RIPA) buffer was used to lyse CRC cells and tissues for 50 min on ice, followed by 15 min of centrifugation of whole‐cell lysates at 15,000 rpm and 4°C. The protein content was determined by the bicinchoninic acid (BCA) assay. Besides, each sample was added with the loading buffer (Beyotime Bio Inc), followed by 5 min of denaturation under 95°C. Western blotting was conducted in accordance with specific protocols.^21^ In the present work, the primary antibodies utilized included anti‐MMP9 (Proteintech, Cat# 10375–2‐AP), anti‐PCNA (CST, Cat# 2586S), anti‐IBSP (CST, Cat# 5468S), anti‐E‐cadherin (CST, Cat# 3195T), anti‐MMP2 (Proteintech, Cat# 10373–2‐AP), anti‐N‐cadherin (CST, Cat#13116T), anti‐Vimentin (CST, Cat# 5741S), anti‐CyclinD1 (CST, Cat# 55506S), anti‐Cdk4 (CST, Cat# 12790T), anti‐Bcl‐2 (CST, Cat# 15071T), anti‐Bax (CST, Cat# 5023S), anti‐Fyn (Abcam, Cat# ab125016), anti‐p‐Fyn (phosphor Y530, Abcam, Cat# ab188319), anti‐β‐catenin (CST, Cat# 8480T), anti‐p‐β‐catenin (phosphor Y142, Abcam, Cat# ab27798), and anti‐β‐actin (Beyotime Bio Inc, Cat#AA128). Besides, the enhanced chemiluminescence (ECL) detection system was employed to detect the protein–antibody complex. Using β‐actin as reference, protein level was quantified by the relative gray value.

### 2.6 Enzyme‐linked immunosorbent assay

The concentrations of IBSP in serums were measured with ELISA Kit (Meimian Biotechnology Co. Ltd) according to the manufacturer's instructions. Data were read by a multi‐detection microplate reader (Tecan) at 450 nm.

### 2.7 Potential biological functions of IBSP according to LinkedOmics analysis

The possible IBSP biological effects were explored against the LinkedOmics database.^22^ The LinkFinder module was utilized for investigating the IBSP‐related DEGs from TCGA CRC cohort (n = 629). We adopted gene set enrichment analysis (GSEA) for GO, KEGG pathway, and kinase‐target enrichment analyses after signing and ranking LinkFinder results at the threshold of FDR < 0.05 after 500 simulations. We also used the clinical parameters and survival information of 586 CRC patients from TCGA data to apply the preliminary study on the relationship between IBSP and survival of CRC patients. R language packages “survival” and “survminer” were used to make Kaplan–Meier survival curve and apply the log‐rank test. According to the median value of IBSP expression, patients were divided into two groups.

### 2.8 Gene sets associated with metastatic recurrence

GSEA was performed using GSE17538 data to identify the gene sets associated with metastatic recurrence (GSEA v2.2.2). Using the ranked gene list, GSEA was carried out based on the Molecular Signatures Database‐derived hallmark gene sets (version 5.1, Broad Institute). This study utilized the GSEA default parameters with the gene lists containing 15–500 genes, and 1000 permutations were run for analysis. The FDR *q* value <0.25 indicated statistical significance.

### 2.9 Tumor promoter function of IBSP

For detecting the effect of IBSP gene in promoting tumorigenesis, this study conducted the colony formation assay after IBSP‐siRNA or IBSP‐overexpressing plasmid transfection. For this experiment, CRC cells (1 × 10^3^) were inoculated into the 6‐well plate and cultured for 10 days. Afterward, we determined the viable colony number (>50 cells/colony) and used Giemsa to stain the colonies for thrice. Besides, the CRC cell growth ratio was determined by CCK8 (Yeasen) assay according to specific protocols. Each experiment was carried out three times.

### 2.10 Cell apoptosis

After IBSP‐siRNA was transfected into CRC cells, their apoptosis was studied according to the Annexin‐V‐fluorescein isothiocyanate (FITC)/propidium iodide (PI) (Yeasen) double‐staining approach. One blank control together with one single staining control using two dyes were set in this assay. Following trypsinization in the absence of ethylenediaminetetraacetic acid (EDTA), all cells were subjected to 5 min of centrifugation at 1000 rpm for removing supernatants. Later, cell pellets were acquired, rinsed by PBS twice, and harvested through centrifugation. Thereafter, 500 μl of binding buffer was used to resuspend cells, followed by the addition of 5 μl of Annexin‐V‐FITC and 5 μl of PI. Then, the cells were stained in dark for 15–20 min and flow cytometric analysis was conducted. The assay was conducted for three times.

### 2.11 Cell cycle experiment

In this assay, the collected cells were washed with PBS for two times then the cell density was adjusted to 1 × 10^6^ cells/ml. After the supernatants were discarded, 500 μl of the 70% pre‐cooled ethanol was added to fix cells overnight under 4°C. After removing ethanol, the cold PBS solution was used to wash cells thrice. Then, we added 500 μl of PI/RNase (Yeasen) staining working solution made in advance to stain cells for 30 min under 4°C, and later flow cytometric analysis was conducted. This assay was repeated for three times.

### 2.12 Transwell assay

After IBSP‐siRNA or IBSP‐overexpressing plasmid transfection, CRC cells were cultivated into the previously coated upper transwell chamber (Corning) then medium containing 20% fetal bovine serum was added into the lower chamber. After 36 h, each transwell chamber was washed with PBS buffer and non‐invasive cells were gently removed using cotton swabs. The remaining invasive cells were fixed in 100% methanol and stained with 0.25% crystal violet for at least 15 min at room temperature. Triplicate readings for each sample were taken at five random sites within each image.

### 2.13 Statistical analysis

Statistical analysis was completed using GraphPad prism8 software and SPSS20.0. The values were presented in the manner of mean ± SD from three independent experiments. Comparisons of two groups were analyzed by student's *t*‐test. Analysis of variance (ANOVA) was used to confirm the comparisons among multiple groups. The survival rate was calculated by log‐rank test and comparisons were confirmed by the Kaplan–Meier method. A difference of *p *< 0.05 indicated statistical significance.

## 3 RESULTS

### 3.1 Identification of metastatic recurrence‐related genes in CRC

**FIGURE 1:**
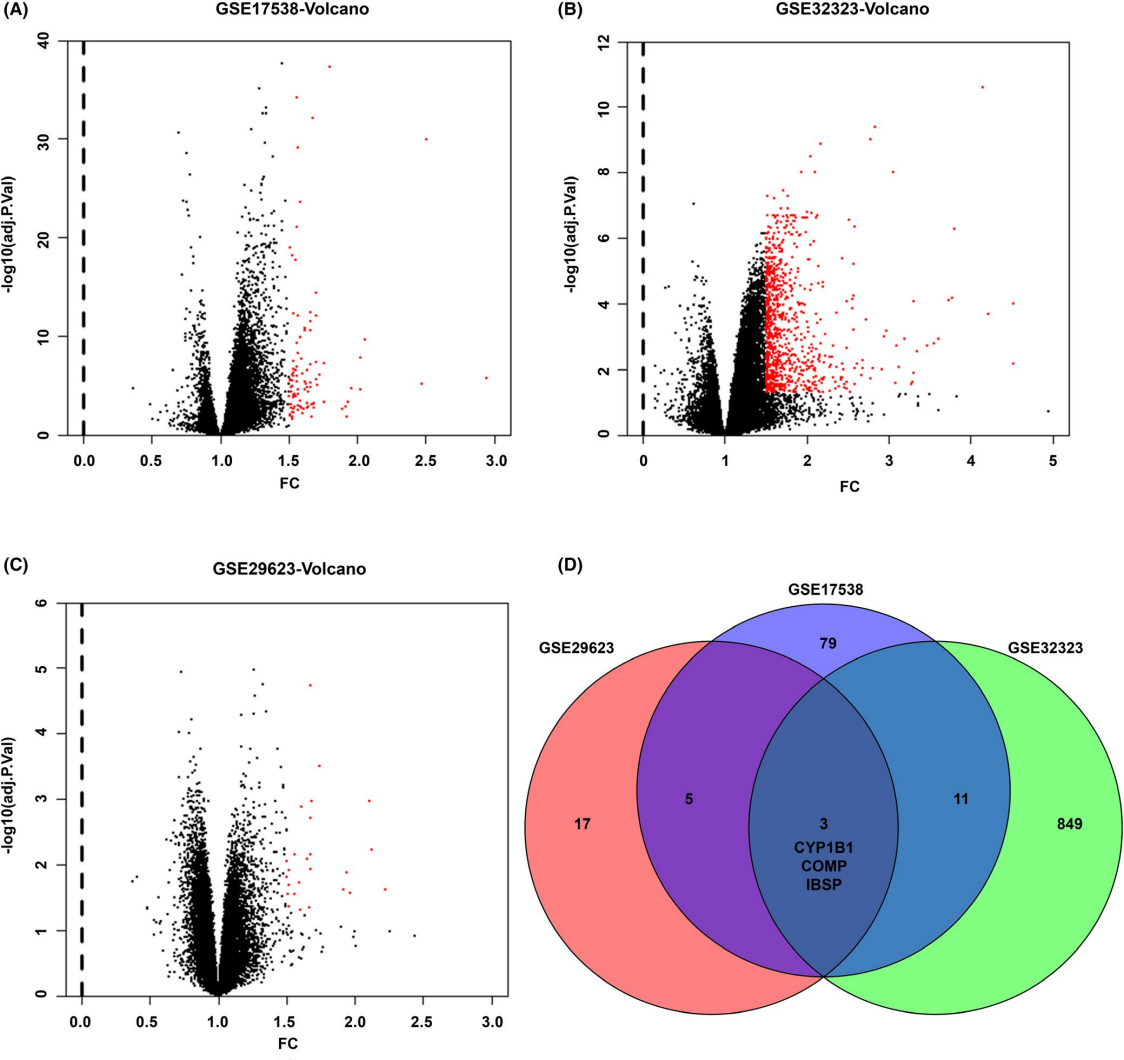
Biomarker discovery analysis in this study. (A) The significantly upregulated genes of patients with recurrence in GSE17538 dataset. (B) The significantly upregulated genes of patients with metastasis or metastatic recurrence in GSE32323 dataset. (C) The significantly upregulated genes of patients with recurrence in GSE29623 dataset. (D) Venn plots of upregulated overlapping genes

Genes specific to metastatic recurrence of CRC were obtained from the public GSE17538, GSE32323, and GSE29623 datasets. The volcano plots were used to visualize the significantly upregulated genes of all the three datasets (>1.5‐fold change, and adjusted *p* < 0.05; Figure 1A–C). The red dots represented significantly upregulated genes. Venn diagram was generated to identify the common overexpressed genes. At last, three significantly upregulated genes (CYP1B1, COMP, and IBSP) were identified (Figure 1D). Since, the clinical significance of IBSP is still poor, yet much attention has been paid in cancer research. Therefore, IBSP was selected in this study for better analysis.

### 3.2 Verification of IBSP expression in several independent datasets, clinical specimens, and CRC cell lines

**FIGURE 2:**
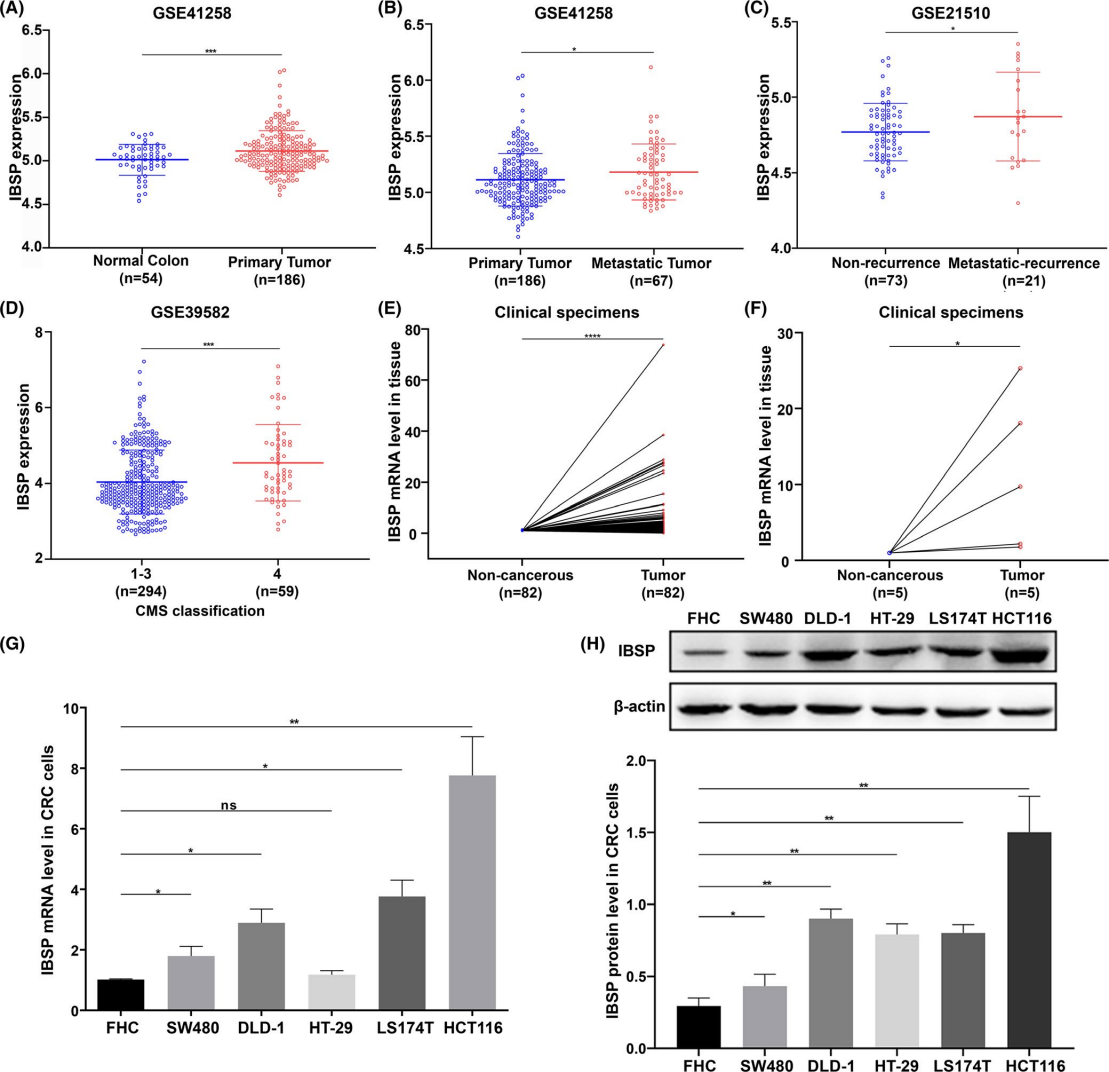
IBSP expression was upregulated in various independent datasets, clinical specimens, and CRC cell lines. *p < 0.05, **p < 0.01, ***p < 0.001, ****p < 0.0001, ns: nonsignificant. (A) and (B) IBSP expression was specifically higher in the primary CRC and metastatic CRC, respectively, compared with normal colonic mucosa and primary CRC in GSE41258 dataset. (C) IBSP expression was specifically higher in CRC patients with metastatic recurrence, compared with CRC patients with non‐recurrence in GSE21510 dataset. (D) IBSP expression was specifically higher in the consensus molecular subtype 4 CRC patients (CMS4, with high risk of recurrence), compared with the CMS1, 2,3 CRC patients (with low risk of recurrence) in GSE39582 dataset. (E) The mRNA level of IBSP was specifically higher in the primary CRC, compared with normal colorectal mucosa in clinical specimens. (F) The mRNA level of IBSP was specifically increased in the CRC liver metastatic tumors, compared with normal liver tissues in clinical specimens. (G and H) The mRNA and protein level of IBSP were significantly increased in several CRC cell lines compared to human normal colorectal epithelial FHC cells

To verify IBSP is associated with metastatic recurrence of CRC, we verified the IBSP expression in several independent datasets. We found that IBSP expression was specifically higher in the primary CRC, metastatic CRC, CRC patients with metastatic recurrence, and high risk of recurrence, respectively, compared with normal colonic mucosa, primary CRC, CRC patients with non‐recurrence, and low risk of recurrence (Figure 2A–D). We obtained consistent results in clinical specimens, compared with adjacent non‐tumor tissues, and the IBSP mRNA and protein levels evidently increased in primary CRC tissue samples (Figure 2E and Figure S1A). Significant association was observed between the mRNA expression of IBSP with the CRC tumor grade and lymph node metastasis (*p *< 0.05, Table 1). Additionally, compared with adjacent non‐tumor tissues, the IBSP mRNA and protein levels also evidently increased in CRC liver metastatic recurrence tumors (Figure 2F and Figure S1B). In addition, the IBSP mRNA and protein levels remarkably increased in DLD‐1, HCT116, SW480, HT‐29, and LS174 T in comparison with FHC cells (Figure 2G–H).

### 3.3 The diagnostic capacity of the IBSP in serums

**FIGURE 3:**
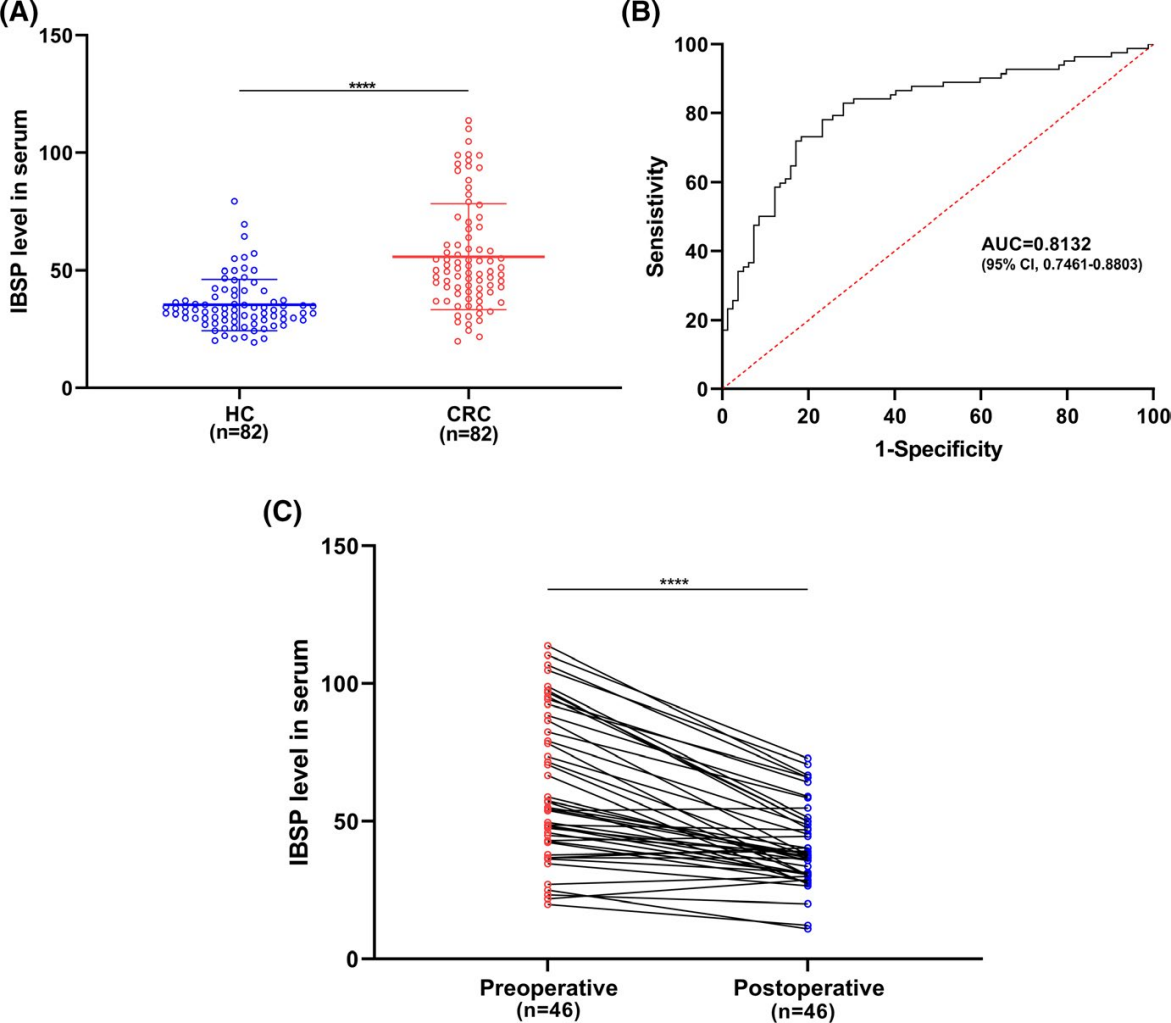
IBSP level in the serum act as a novel potential diagnostic biomarker for CRC. (A) IBSP level in the serum was significantly higher in CRC patients compared to healthy controls. HC: healthy controls, **** *p* < 0.0001. (B) The AUC was 0.8132 (95% CI, 0.7461–0.8803) of IBSP for distinguishing CRC patients from healthy controls. (C) IBSP level in the serum of CRC patients was significantly downregulated after surgical resection, suggesting that IBSP might be an indicator of prognosis monitoring.**** *p* < 0.0001

The relative level of serum IBSP evidently elevated among CRC patients relative to normal controls (Figure 3A). For assessing IBSP for its possible diagnostic significance, we performed ROC analysis and determined the AUC value. According to our results, the AUC value in distinguishing CRC cases from normal subjects was determined to be 0.8132 (95% CI, 0.7461–0.8803) (Figure 3B). Besides, ELISA was conducted to determine the serum IBSP levels before and after surgery in certain CRC cases. The serum IBSP level markedly declined after surgery relative to before surgery (Figure 3C). According to the above findings, IBSP might serve as a candidate biomarker to predict the diagnosis and prognosis of CRC.

### 3.4 Biological interaction network and clinical significance of IBSP in CRC

**FIGURE 4:**
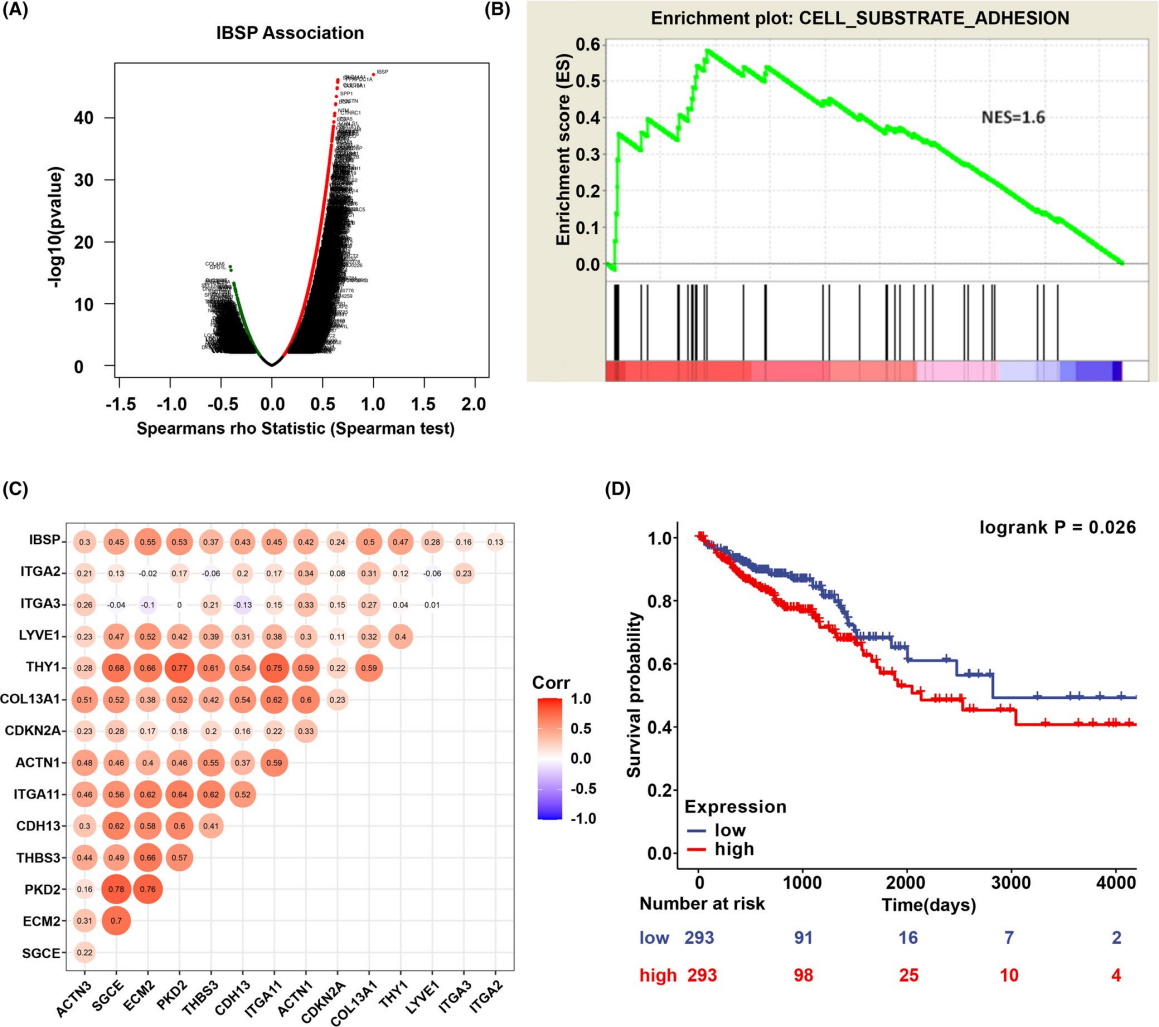
Biological interaction network and clinical significance of IBSP in CRC. (A) The correlations between IBSP and the differentially expressed genes in CRC. (B) According to the GSEA in GSE17538 dataset, cell‐matrix adhesion gene set was correlated with metastatic recurrence in CRC. (C) The correlation of IBSP and representative cell‐matrix adhesion‐related genes in the GSE17538 dataset. (D) IBSP expression was correlated with survival of CRC patients. Low expression of IBSP conferred a survival advantage to CRC patients

For better understanding of IBSP for its molecular role within CRC, we utilized the function module in LinkedOmics for analyzing the mRNA sequencing profiles of 629 TCGA‐derived CRC cases. The volcano plot showed the correlations between IBSP and the differentially expressed genes in CRC (Figure 4A) and the top 50 positive and negative IBSP gene sets were presented in the heat map (Figure S2A). As suggested by our results, IBSP significantly affected the transcriptome. GSEA on TCGA data indicated that the IBSP‐related genes were mostly located in the cell adhesion molecules (CAMs), Wnt‐protein binding, growth factor binding, collagen binding, fibronectin binding, integrin‐mediated signaling pathway, substrate‐dependent cell migration, and so on (Figure S2B). Next, we performed GSEA in GSE17538 dataset to identify the gene sets correlated with metastatic recurrence. The cell‐matrix adhesion gene set showed potent association with metastatic recurrence according to the normalized enrichment score (NES) (Figure 4B). Several genes associated with cell‐matrix adhesion also showed tight correlation with IBSP (Figure 4C), suggesting that IBSP might be used to be the vital predictor for cell‐matrix adhesion and metastatic recurrence phenotype in CRC. For exploring the association of IBSP level with patient prognosis, we extracted the clinical parameters and survival data of 586 CRC patients from TCGA database and Kaplan–Meier survival curve was plotted. According to the median value of IBSP expression, we divided the patients into two groups: high expression and low expression separately represented by red and blue. Low IBSP expression conferred a survival advantage to CRC patients (Figure 4D).

### 3.5 IBSP downregulation suppressed the proliferation and promoted the apoptosis and cell cycle arrest of CRC cells

**FIGURE 5:**
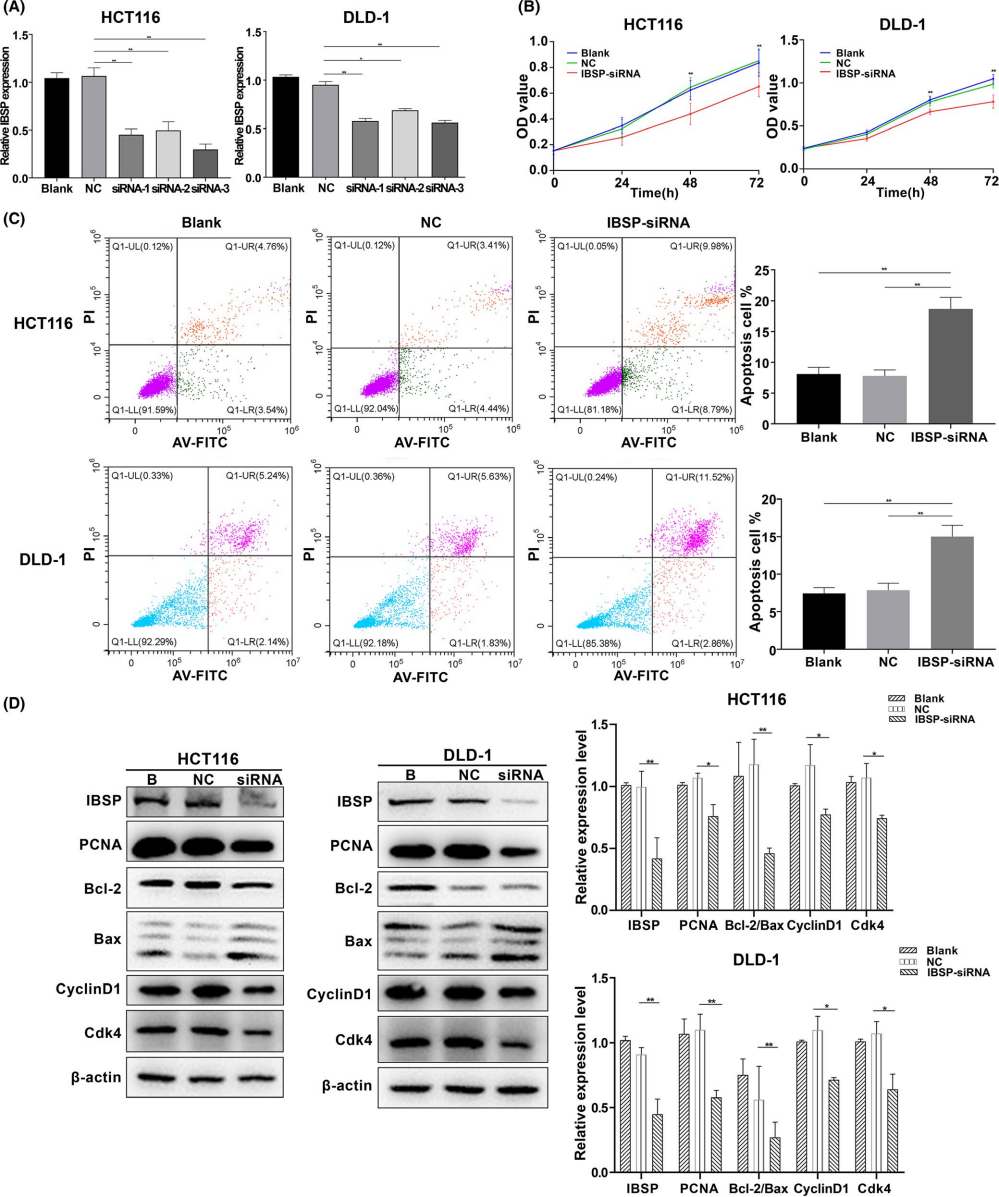
IBSP‐siRNA inhibited proliferation and induced apoptosis in CRC cells. B: blank, NC: negative control, *p < 0.05, **p < 0.01. (A) Downregulation of IBSP mRNA in CRC cells. (B) IBSP‐siRNA inhibited cell proliferation of CRC cells. (C) IBSP‐siRNA increased apoptosis of CRC cells. (D) The protein level of PCNA, Bcl2/Bax, Cyclin D1, and Cdk4 in CRC cells decreased after transfection with IBSP‐siRNA

For assessing the IBSP biological effect within CRC, IBSP expression was downregulated using siRNAs in HCT116 and DLD‐1 cells in which IBSP expression was high according to the Figure 2H. The mRNA and protein level were both downregulated after IBSP‐siRNA transfection (Figure 5A and Figure S3A). siRNA‐3 had the best inhibitory effect on IBSP expression and was used to perform the follow‐up experiments. The above cells were utilized to investigate their proliferation, cell cycle, and apoptosis following IBSP downregulation. According to the results of CCK8 and colony formation assays, the proliferation of cells significantly decreased following transfection with IBSP‐siRNA (Figure 5B and Figure S3B). Flow cytometry confirmed that apoptosis and proportion of cells at G0/G1 phase clearly elevated after IBSP downregulation, while those of cells at S and G2/M phases markedly decreased (Figure 5C and Figure S3C). In addition, the protein level of Bcl2, PCNA, Cdk4, and Cyclin D1 were downregulated, whereas Bax level was upregulated (Figure 5D). The above findings indicated that, IBSP downregulation suppressed the proliferation of cells, induced their apoptosis, and caused cell cycle arrest at G0/G1 phase.

### 3.6 IBSP downregulation inhibits invasion, migration, and epithelial–mesenchymal transition (EMT) of CRC cells

**FIGURE 6:**
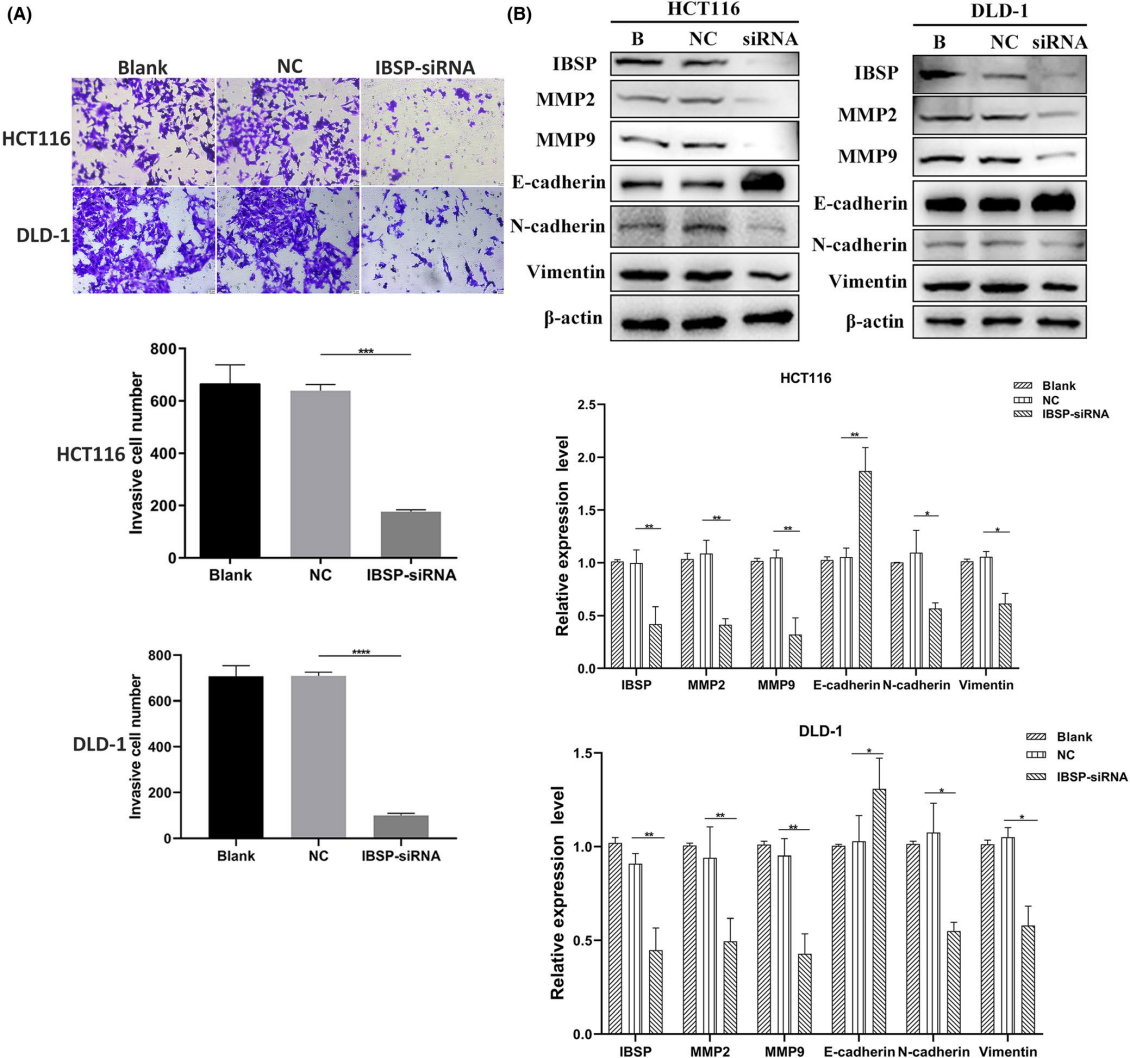
IBSP‐siRNA inhibited invasion, migration, and epithelial–mesenchymal transition (EMT) of CRC cells. *p < 0.05, **p < 0.01, ***p < 0.001,**** *p* < 0.0001. (A) IBSP‐siRNA suppressed invasion and migration of CRC cells. (B) Transfection of IBSP siRNA decreased the protein level of MMP2, MMP9, N‐cadherin, and Vimentin and increased the expression of E‐cadherin

This study performed transwell analysis for measuring the alterations of cell invasion following the downregulated expression of IBSP. As discovered by our results, the HCT116 and DLD‐1 cell lines exhibited markedly declined migration and invasion capacities after the downregulated expression of IBSP (Figure 6A). Western blotting analysis showed that MMP2, MMP9, Vimentin, and N‐cadherin levels declined, whereas E‐cadherin level elevated (Figure 6B). Such results suggested that IBSP downregulation suppressed CRC cell migration, invasion, as well as the EMT process.

### 3.7 IBSP promoted the CRC development via activation of Fyn/β‐catenin signaling pathway

**FIGURE 7:**
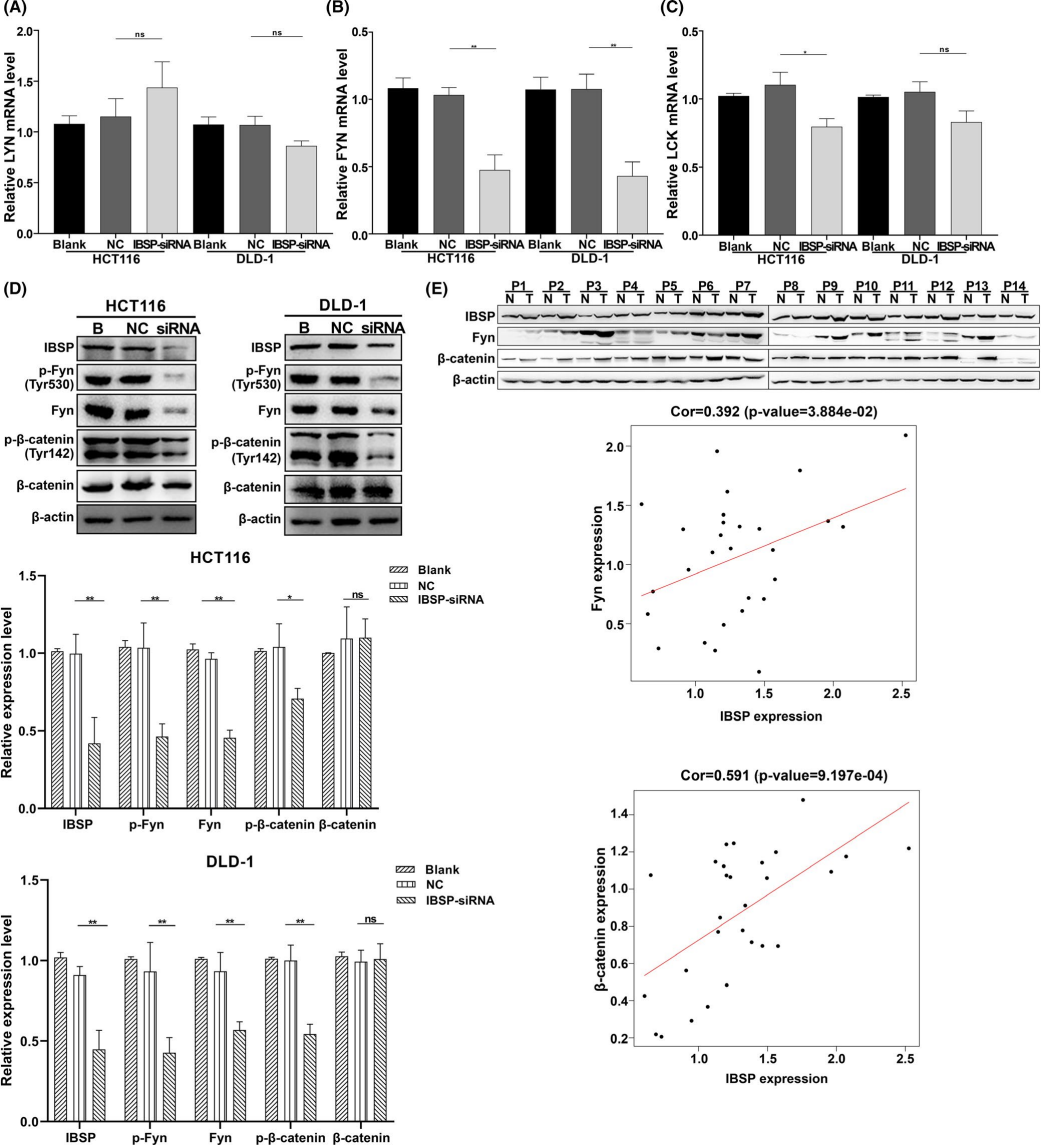
The mechanisms of IBSP function in CRC. *p < 0.05; **p < 0.01; ns: nonsignificant. (A) The LYN mRNA expression following IBSP downregulation. (B) The FYN mRNA expression following IBSP downregulation. (C) The LCK mRNA expression following IBSP downregulation. (D) Protein level of Fyn, p‐Fyn, and p‐β‐catenin decreased after transfection of IBSP‐siRNA, while β‐catenin showed no significant variation. (E) The expression of IBSP was significantly positively correlated with the expression of Fyn and β‐catenin

For better exploring the IBSP targets in CRC, the kinase networks of GSEA‐derived gene sets with positive correlation were analyzed. Typically, the top five kinase‐target networks were mainly associated with the lck/yes‐related novel protein tyrosine kinase (LYN), tyrosine‐protein kinase fyn (FYN), lymphocyte cell‐specific protein‐tyrosine kinase (LCK), protein tyrosine kinase 2 (PTK2), and protein kinase C beta (PRKCB) (Table 4). According to the results from qRT‐PCR, FYN mRNA expression decreased in both HCT116 and DLD‐1 cells after transfection of IBSP‐siRNA (Figure 7A–C). Fyn and p‐Fyn protein expression also decreased following IBSP downregulation (Figure 7D). Previous studies have shown that Fyn phosphorylate β‐catenin at Tyr142 site and promote the dissociation of E‐cadherin–catenin complex.^23^ Western blotting analysis showed that following IBSP downregulation, the level of p‐β‐catenin decreased in both cell lines, while expression of β‐catenin showed no significant variation (Figure 7D). We also detected the expression of IBSP, Fyn, and β‐catenin in tumor and non‐carcinoma tissues of 14 patients and calculated their correlation. The expression of IBSP was significantly positively correlated with the expression of Fyn (Cor = 0.392, *p* = 0.03884) and β‐catenin (Cor = 0.591, *p *= 0.0009197) (Figure 7E). These results indicate that IBSP promoted the CRC development via activation of Fyn/β‐catenin signaling pathway.

**TABLE 4:** The kinase regulatory network of IBSP in CRC (LinkedOmics)

Enriched category	Gene set	Leading edge number	*p* value
Kinase target	Kinase_LYN	18	0.0020284
	Kinase_FYN	18	0.0020704
	Kinase_LCK	17	0.0040486
	Kinase_PTK2	6	0.013636
	Kinase_PRKCB	25	0.018145

### 3.8 IBSP overexpression promoted CRC cell proliferation, invasion as well as migration via Fyn/β‐catenin pathway

**FIGURE 8:**
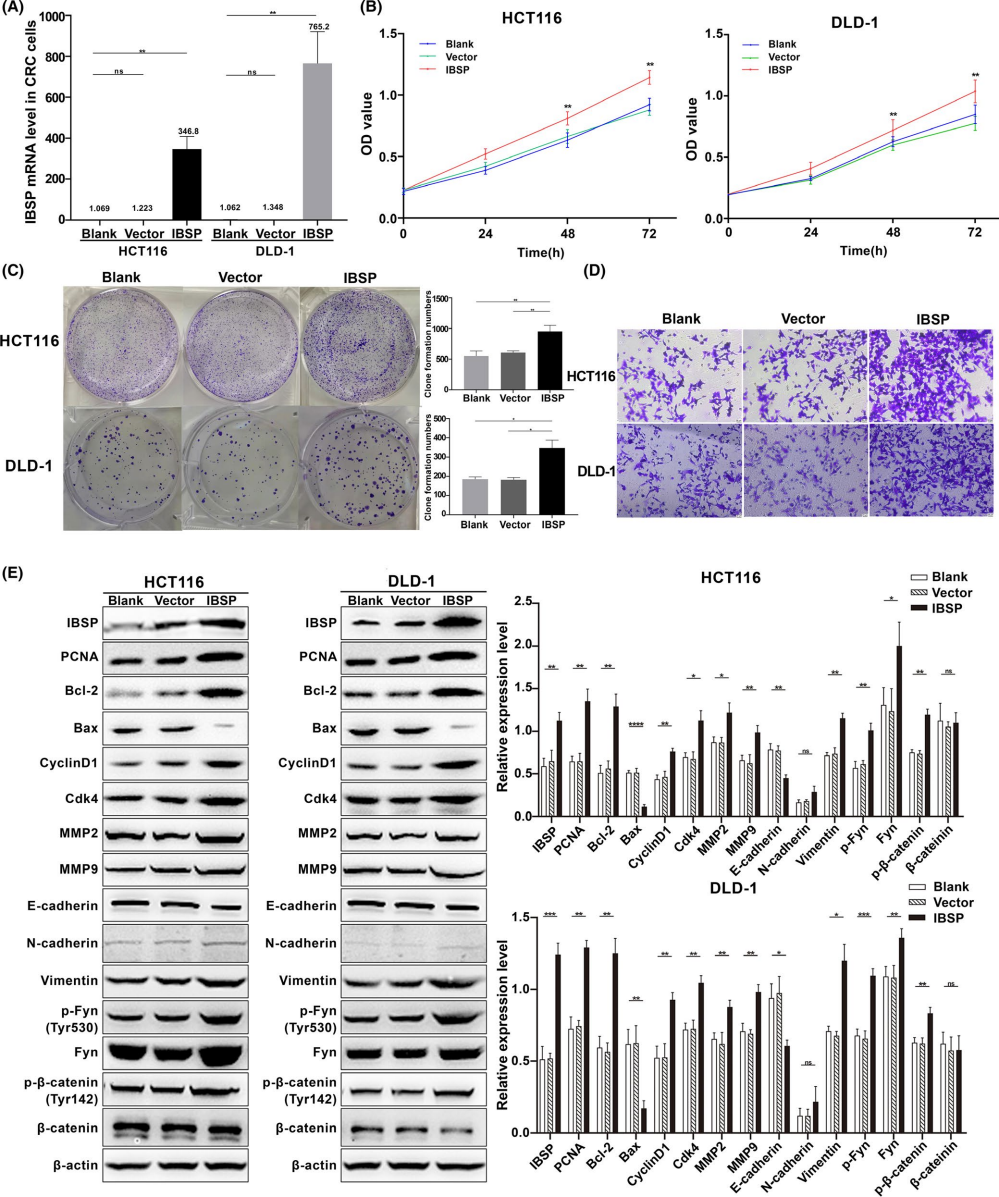
IBSP overexpression promoted CRC cell proliferation, invasion as well as migration via Fyn/β‐catenin pathway. *p < 0.05, **p < 0.01, ***p < 0.001,**** *p* < 0.0001, ns: nonsignificant. (A) Upregulation of IBSP mRNA in CRC cells. (B) IBSP overexpression promoted cell proliferation of CRC cells. (C) IBSP overexpression significantly increased the number of cell colonies. (D) IBSP overexpression promoted invasion and migration of CRC cells. (E) IBSP overexpression increased the protein level of PCNA, Bcl2, Cyclin D1, Cdk4, MMP2, MMP9, N‐cadherin, Vimentin, Fyn, p‐Fyn, and p‐β‐catenin and decreased the expression of Bax and E‐cadherin

Furthermore, we overexpressed IBSP in HCT116 and DLD‐1 cells (Figure 8A) to further verify the function of IBSP. As shown in Figure 8B–E, overexpression of IBSP significantly promoted proliferation, invasion as well as migration of CRC cells, and the changes of related proteins were also detected by Western blotting. In addition, the overexpression of IBSP also resulted in the upregulation of Fyn, p‐Fyn, and p‐β‐catenin. Therefore, our data consistently suggested that a high level of IBSP expression promotes the growth and aggressiveness of CRC cells.

## 4 DISCUSSION

Metastasis and recurrence are the main causes of cancer mortality. In this study, we identified and verified IBSP to be a metastatic recurrence‐related gene and a potential valuable marker for the diagnosis and prognosis of CRC. IBSP belongs to the SIBLING family that contains dental matrix protein, osteopontin, extracellular phosphoglycoprotein along with salivary phosphate protein as well.^24^, ^25^, ^26^ A majority of SIBLING family members can be primarily detected within bone tissues, a small portion of these proteins show abnormal expression within malignant cancer tissues.^18^, ^27^ The aberrantly expressed IBSP gene is tightly associated with an increased risk of malignant transformation, bone metastasis, along with dismal prognostic outcome of non‐small cell lung cancer (NSCLC), BC, and PC.^15^, ^17^, ^28^, ^29^, ^30^ There are few reports on the relationship between IBSP and CRC, only a study of regulation of osteopontin and related proteins in rat CC531 CRC cells.^31^ The IBSP expression in human CRC cells and clinical specimens was never reported and the biological function and mechanism of IBSP in CRC are still completely unknown. As far as we know, this is the first time to report mRNA and protein level of IBSP was upregulated in CRC cells and patients and to study the biological function of IBSP and possible signaling pathway of IBSP involved in CRC progression. Our results showed that overexpression of IBSP significantly promoted proliferation and migration of CRC cells and downregulation of IBSP evidently suppressed CRC cell proliferation, migration as well as invasion, but promoted their apoptosis together with the arrest of cell cycle.

GSEA in GSE17538 dataset showed that the cell‐matrix adhesion gene set was strongly correlated with metastatic recurrence of CRC. The genes in the cell‐matrix adhesion gene set were strongly correlated with IBSP, suggesting that IBSP expression may serve as an important indicator of cell‐matrix adhesion and metastatic recurrence phenotype in CRC. The adhesion between cells, and between cells and matrix plays a vital part in the function and differentiation of epithelial cells. Typically the adhesion between cells and extracellular matrix (ECM) exerts a key role in maintaining tissue integrity as well as human health.^32^ IBSP is a binding molecule that exerts a critical part during protein‐migrating cell surface adhesion. The adhesive plaques can stimulate molecular signal formation, which can thus boost transfer factor precursor formation. ^33^ Under normal condition, IBSP can interact with integrins, the primary cell adhesion receptors involved in almost all cancer progression processes.^34^, ^35^ IBSP can also act on integrin ligand, an RGD sequence at the proximal protein C‐terminal with high conservation degree, thus enhancing cell adhesion.^36^, ^37^ IBSP can form a three‐molecule complex with avb3 integrin as well as matrix metalloproteinase 2 (MMP2), and the complex accelerates local matrix decomposition along with cancer cell invasion.^37^, ^38^ According to our study, MMP2 was downregulated following IBSP‐siRNA transfection.

This work presented that IBSP was related to EMT. Cancer cells can obtain their metastasis potential through EMT, which may promote their migration and invasion and exerts a vital part in their malignant transformation.^39^ The upregulated expression and strengthened effect of N‐cadherin within epithelial cells, or the weakened effect and downregulated expression of E‐cadherin have been identified as the markers of EMT.^40^, ^41^, ^42^ According to our Western blotting analysis, N‐cadherin was downregulated and E‐cadherin was upregulated following IBSP‐siRNA transfection, which indicated that EMT was possibly related to the IBSP effect on the proliferation of CRC cells. GSEA showed that Fyn might be kinase target of IBSP and our results verified that the β‐catenin and Fyn phosphorylation degrees were, respectively, downregulated and upregulated after transfection with IBSP‐siRNA and IBSP‐overexpressing plasmid, suggesting that IBSP promoted the CRC development via activation of Fyn/β‐catenin signaling pathway. There is almost no free β‐catenin in the cytoplasm of normal colonic epithelium, β‐catenin accumulates and evades the proteasomal destruction complex, translocates into the nucleus, interacts with lymphoid enhancer factor/T‐cell factor (LEF/TCF) factors, induces the transactivation of target genes involved in cell proliferation, apoptosis, adhesion and angiogenesis, such as c‐myc, CyclinDl, MMP7, survivin as well as CD44, and promotes the occurrence and metastasis of tumor.^43^, ^44^, ^45^ Previous studies have shown that Fyn phosphorylate β‐catenin at Tyr142 site and promote the dissociation of E‐cadherin–catenin complex, leading to cell–cell adhesion loss regulated by cadherin, along with increased β‐catenin expression in cytoplasm.^46^, ^47^, ^48^ The knockdown of IBSP decrease the phosphorylation of Fyn and β‐catenin, stabilize the binding of E‐cadherin–catenin complex, enhance the adhesion between cells mediated by E‐cadherin, reduce the free β‐catenin level in cytoplasm as well as inhibit the proliferation and invasion of cancer cells.

We should point out the limitations of the present study. First, the relationship between IBSP and metastatic recurrence as well as diagnostic capacity of the IBSP were only evaluated in a clinical cohort with small sample size, further expansion of samples size is necessary. Second, we identified and validated IBSP was associated with metastatic recurrence of CRC in some patients and in vitro models, there remains a need for in vivo study to demonstrate IBSP to be a key gene associated with metastatic recurrence of CRC. Finally, the mechanisms how IBSP is involved in cell proliferation, apoptosis, cell cycle, invasion, migration, and EMT might be the abnormal activation of Fyn/β‐catenin signaling pathway, the detailed mechanism of IBSP and Fyn/β‐catenin signaling pathway involved in CRC progression will be clarified in the follow‐up research.

In summary, we identified and validated IBSP to be a key gene associated with metastatic recurrence of CRC and a potential valuable marker for the diagnosis, treatment, and prognosis monitoring of CRC. The mRNA and protein level of IBSP were upregulated in CRC cells and patients. The relative level of serum IBSP evidently increased among CRC patients relative to normal controls, and downregulated after operation. IBSP upregulation was also related to poor survival in CRC. Functional experiments in vitro showed that IBSP promoted the growth and aggressiveness of CRC, and the potential mechanism by which IBSP promoted carcinogenesis of CRC was the abnormal activation of Fyn/β‐catenin signaling pathway. Hopefully, this study can shed more lights on the pathogenic mechanism, diagnosis, and therapeutic strategy for CRC.

## CONFLICT OF INTEREST

The authors have no potential conflict of interest to disclose.

## ETHICAL STATEMENT

This study was approved by the Ethics Committee of Tsinghua Shenzhen International Graduate School. All subjects gave written informed consent in accordance with the Declaration of Helsinki before their inclusion in the study.

## Supporting information

Fig S1‐S3

## Data Availability

The data supporting the results of this study are available in GEO and TCGA database. This data were derived from the following public resources: https://www.ncbi.nlm.nih.gov/gds, http://cancergenome.nih.gov, http://www.cbioportal.org, and https://portal.gdc.cancer.gov.
