# Supplementary material for: IBSP, a potential recurrence biomarker, promotes the progression of colorectal cancer via Fyn/β‐catenin signaling pathway
Source: Cancer Med. 2021 May 13;10(12):4030–45. doi: 10.1002/cam4.3959 (PMC8209559; doi:10.1002/cam4.3959)
Supplement: Supplementary file 1 — Fig S1‐S3 [file CAM4-10-4030-s001.doc]

**Data Supplement**

**Title:**

**IBSP, a potential recurrence biomarker, promotes the progression of colorectal cancer via Fyn/β-catenin signaling pathway**

**Figure S1. The protein level of IBSP in tissues.**

**Figure S2. Biological interaction network of IBSP in CRC.**

**Figure S3. IBSP-siRNA reduced number of cell colonies and induced cell cycle arrest in CRC cells.**


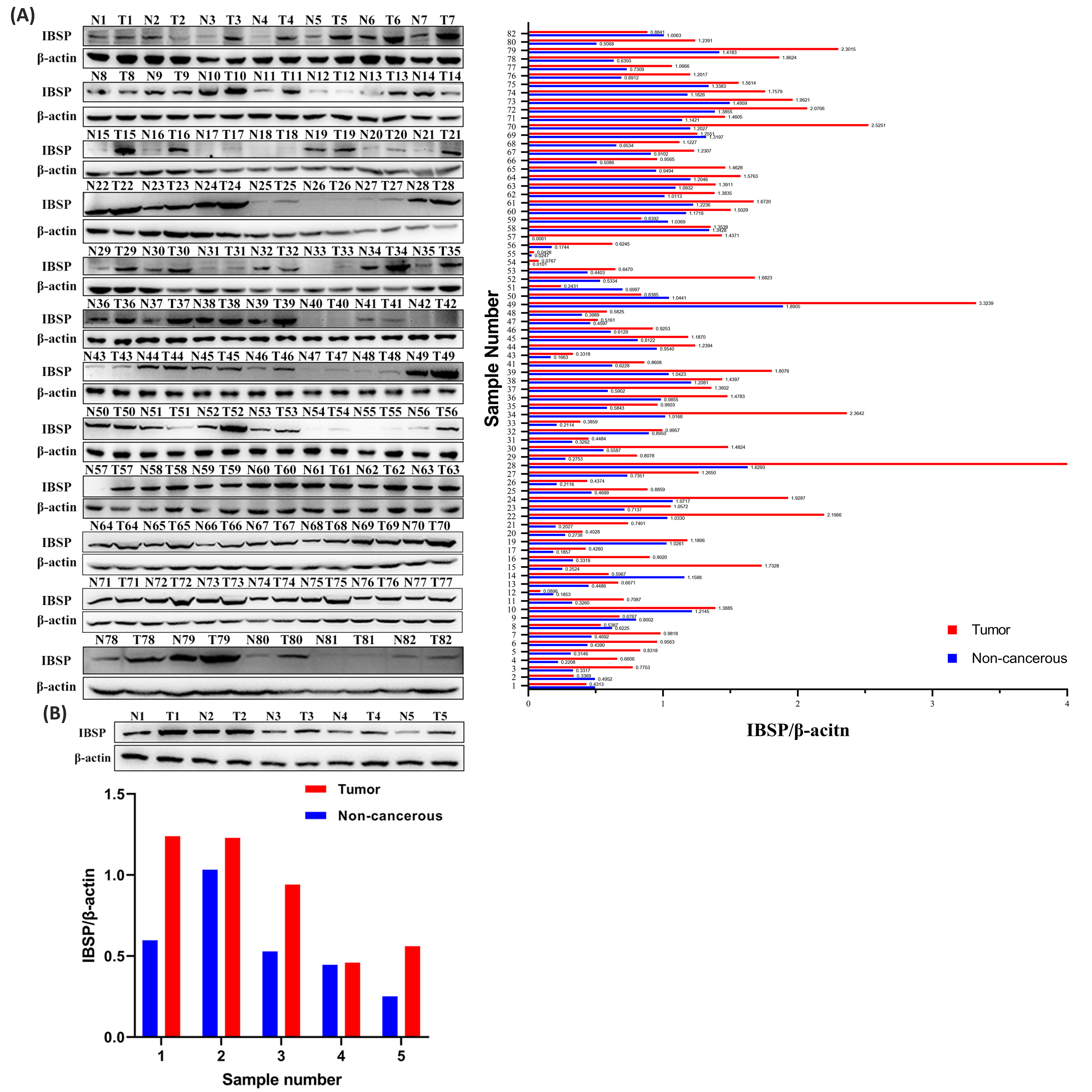


**Figure S1. The protein level of IBSP in tissues.**

A.The protein level of IBSP was specifically higher in the primary CRC, compared with normal colorectal mucosa in clinical specimens.

B.The protein level of IBSP was specifically increased in the CRC liver metastatic tumors, compared with normal liver tissues in clinical specimens.


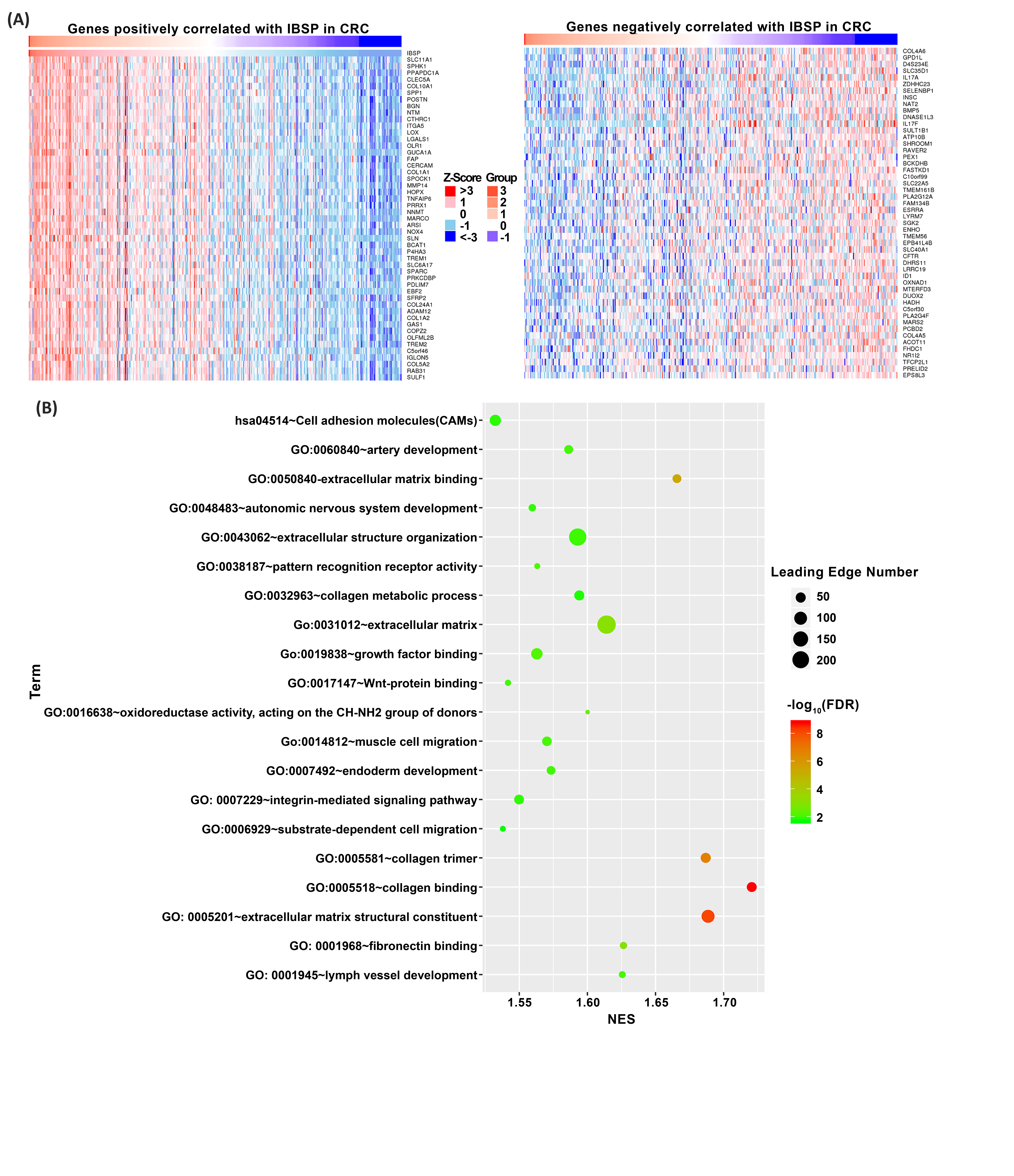


**Figure S2. Biological interaction network of IBSP in CRC.**

A. Heat maps showing the top 50 genes positively and negatively correlated with IBSP in CRC.

B.The significantly enriched GO annotations and KEGG pathways of IBSP co-expression genes in CRC by using GSEA. The top 20 was gathered according to the FDR.


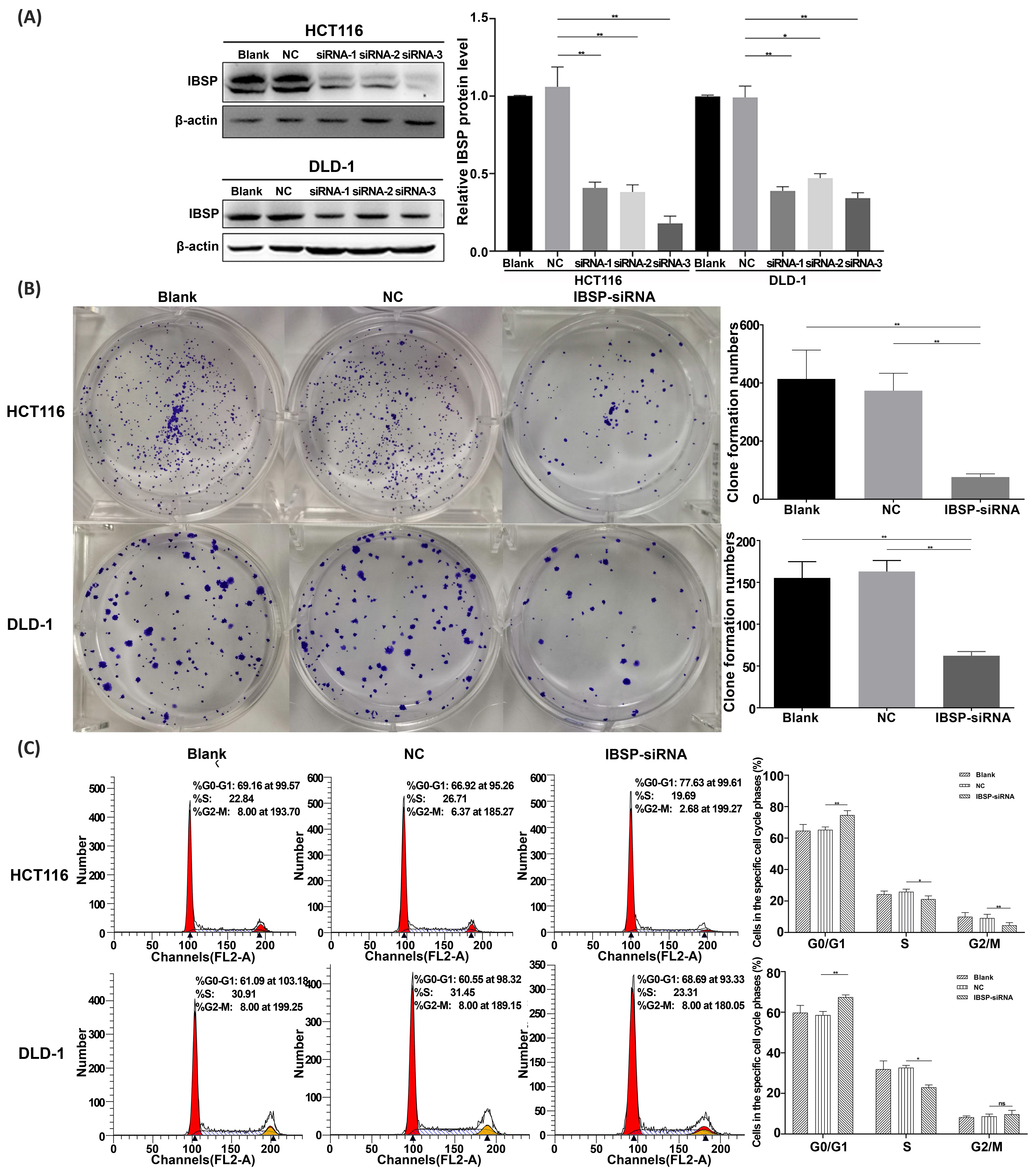


**Figure S3. IBSP-siRNA reduced number of cell colonies and induced cell cycle arrest in CRC cells.** B:blank, NC: negative control, *P < 0.05, **P < 0.01, ns:no significant.

A. Downregulation of IBSP protein.

B. IBSP-siRNA significantly reduced number of cell colonies.

C. G0/G1 phase arrest of CRC cells after IBSP siRNA transfection.
